# Exploring the potential human pathogenic bacteria in selected ready‐to‐eat leafy greens sold in Dhaka City, Bangladesh: Estimation of bacterial load and incidence

**DOI:** 10.1002/fsn3.3825

**Published:** 2023-11-22

**Authors:** Raihan Ferdous, Nazneen Sultana, Md. Belal Hossain, Rifat Ara Sultana, Sanzida Hoque

**Affiliations:** ^1^ Department of Plant Pathology Sher‐e‐Bangla Agricultural University Dhaka Bangladesh

**Keywords:** CFU, contamination, food‐poisoning, incidence, PCR, quality, ready‐to‐eat, safety

## Abstract

This study was designed to investigate the presence of potential human pathogenic bacteria, bacterial load, and their incidence in ready‐to‐eat leafy greens viz., coriander, lettuce, and mint leaves sold at diverse marketplaces in Dhaka City. Multiple identification methods including cultural, morphological, biochemical, and molecular analysis were employed in the Plant Pathology Laboratory of Sher‐e‐Bangla Agricultural University to identify the human pathogenic bacteria. In molecular analysis, the DNA samples were put through PCR using bacterial primer 27F: AGAGTTTGATCMTGGCTGAG and universal primer 1942R: CGGTTACCTTGTTACGACTT. Initially, nine different bacterial genera viz. *Bacillus, Escherichia, Pseudomonas, Neisseria, Klebsiella, Enterobacter, Shigella, Vibrio*, and *Staphylococcus* were detected, and their incidence was 93%, 67%, 44%, 30%, 26%, 26%, 11%, 7%, and 7% respectively. A total of twelve bacteria have been identified from these genera out of which 7 bacteria viz. *Bacillus cereus, Escherichia coli, Pseudomonas aeruginosa, Klebsiella pneumoniae, Enterobacter aerogenes*, *Staphylococcus aureus*, and *Shigella* spp., were reported as human pathogenic bacteria in several pieces of literature. The highest colony‐forming units per gram were shown in mint (4.27 ± 2.35 × 10^9^) followed by lettuce (2.87 ± 0.76 × 10^9^) and coriander (2.43 ± 1.32 × 10^9^). Considering marketplaces, the highest colony‐forming units per gram were observed in the samples of street markets (5.0 ± 1.72 × 10^9^) and the lowest was in supermarkets (1.87 ± 0.46 × 10^9^) followed by local markets (2.7 ± 0.91 × 10^9^). All the leafy green samples crossed the acceptable level of bacterial load (10^6^ CFU/g). The findings of the study highlight the urgency for improved food safety protocols in their production and distribution in Dhaka city.

## Graphical abstract

In this study, nine different bacterial genera were detected in ready‐to‐eat leafy greens viz., coriander, mint, and lettuce leaves. It revealed that the incidence of the detected bacterial genera in ready‐to‐eat leafy greens was as follows: *Bacillus* (93%), *Escherichia* (67%), *Pseudomonas* (44%), *Neisseria* (30%), *Klebsiella* (26%), *Enterobacter* (26%), *Shigella* (11%), *Vibrio* (7%), and *Staphylococcus* (7%).

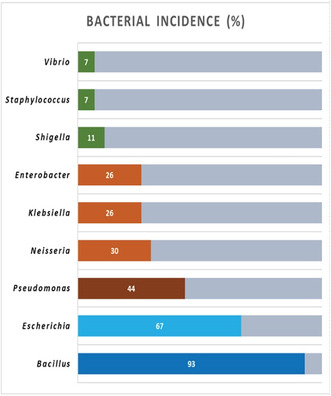

## 1 INTRODUCTION

Leafy greens have excellent nutritional value in the diet and can be used for medicinal benefits. Different phytochemicals are present in leafy greens including phenolic acids, flavonoids, carotenoids, polyphenols, glucosinolates, isothiocyanate, allylic sulfides, phytosterols, and monoterpenes. Leafy greens mostly contain antioxidants, dietary fibers, minerals, α‐linoleic acid, and vitamins. It has different health benefits such as anti‐diabetic properties, preventing CVD, anti‐hypertensive, anti‐carcinogenic, anti‐anemic, and improving gut health (Aslam et al., 2020).

Leafy greens consumption may have health benefits, but recent reports of infectious disease outbreaks from the Centers for Disease Control and Prevention (CDC), US Food and Drug Administration (FDA), World Health Organization (WHO), and Center for Science in the Public Interest (CSPI) have raised questions about the safety and quality of vegetables (Nithya & Babu, 2017). Over the past 20 years, more ready‐to‐eat (RTE) leafy greens have been consumed, and at the same time, more outbreaks of foodborne illness have been linked to the intake of leafy green vegetables (Castro‐Ibanez et al., 2017; Olaimat & Holley, 2012). In such outbreaks, poor sanitation practices were invariably mentioned. The use of chemical fertilizers, hormones, pesticides, and their residues in food are additional possible risks that could raise the risk of foodborne illnesses linked to fresh produce (Chen, 2007).

RTE leafy greens are a convenient approach to ensuring the consumption of vegetables, but consumers must feel convinced that the food is safe to intake. Due to RTE leafy greens are consumed raw and there is no killing step of pathogens (e.g., heating) at any stage in the chain to prevent transmission so the contaminated pathogenic bacteria are still alive after consumption. Therefore, ensuring the microbiological safety of fresh RTE leafy greens offers a special difficulty, and the consumers might get sick if they intake contaminated leafy greens. (Gil et al., 2015).

Fresh leafy greens are generally known to have significant bacterial populations, some of which may be plant endophytes, plant pathogens, or human pathogens (Leff & Fierer, 2013). Several pathogens, like *Salmonella, Shigella, Escherichia coli, Campylobacter, Yersinia, Listeria, Staphylococcus, Bacillus cereus, Vibrio* spp., etc., pose a significant risk to public health, particularly with the consumption of raw produce. According to the Centers for Disease Control and Prevention (2022), and World Health Organization (1998), efforts have been made to address the concern of vulnerabilities arising from the cultivation of leafy greens, which continue to be susceptible to contamination by bacteria like *E. coli* O157, *Salmonella, Shigella, Listeria*, etc., despite attempts at washing.

Ahamad (2020) released a survey report conducted by the Department of Tourism in 2017 found that around 6 million people in Dhaka city consume street food every day which highlighted the concerns regarding hygiene practices surrounding street food preparation and consumption. These street foods, frequently prepared with leafy greens like lettuce in Burgers, coriander in Noodles, and mint in Juices, and served in unsanitary urban environments, stand vulnerable to microbial contamination, as suggested by both the lack of hygiene measures during their preparation and the bustling urban context in which they are sold.

The current study first aims to isolate and identify the range of bacteria present on the surfaces of selected leafy greens, and then, the study aims to specifically detect any human pathogenic bacteria among the isolated bacterial species. Lastly, the research intends to quantitatively assess the colony‐forming units and overall bacterial incidence on these leafy greens to shed light on the safety and hygiene of consuming foods prepared by leafy greens.

## 2 MATERIALS AND METHODS

### 2.1 Study site and period

**FIGURE 1:**
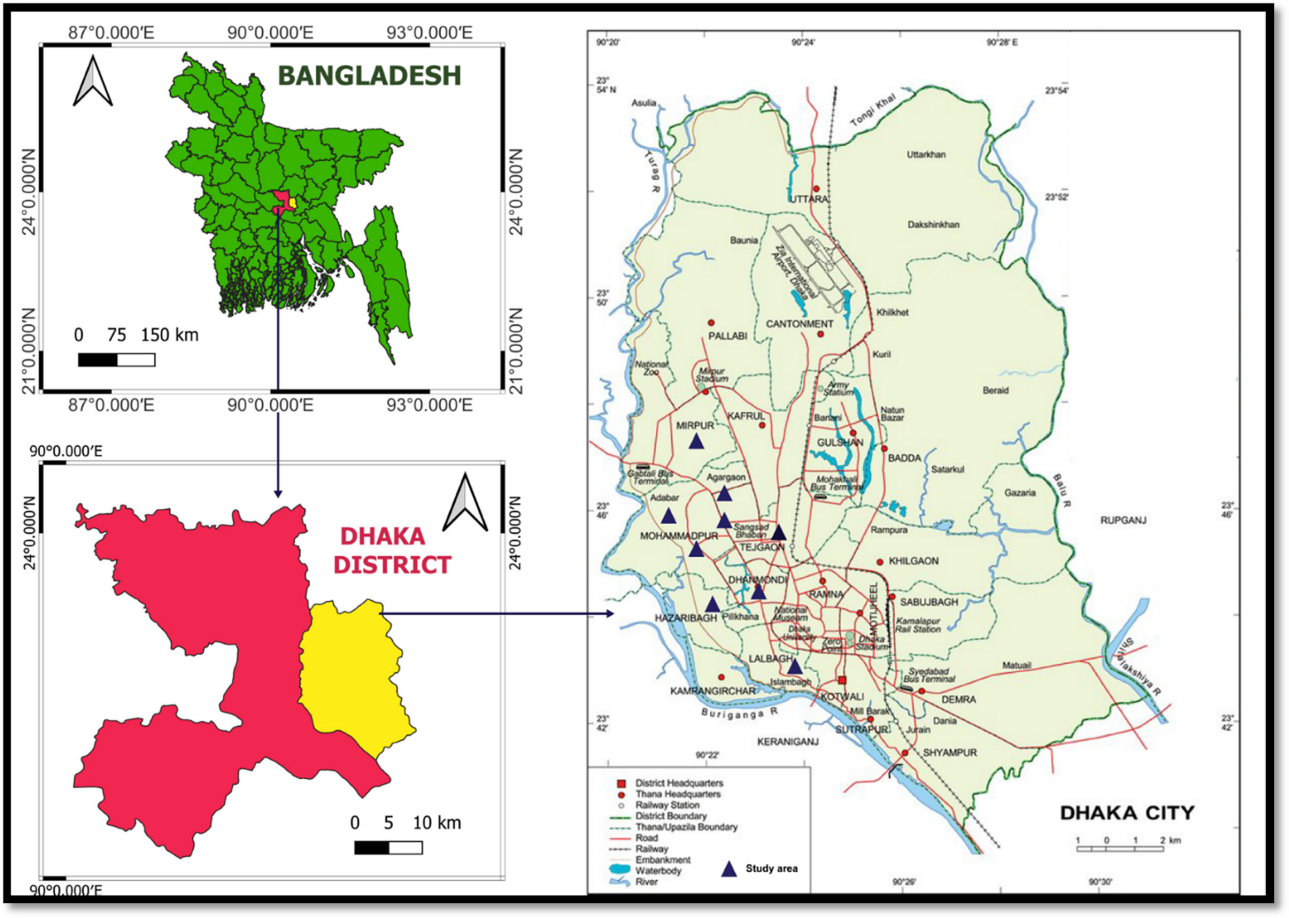
MAP of the study area and locations of the samples collected sites in Dhaka metropolitan city.

The study was carried out in the MS lab of the Department of Plant Pathology at Sher‐e‐Bangla Agricultural University (23°46′13.68″ N, 90°22′39.42″ E) Sher‐e‐Bangla Nagar, Dhaka‐1207, during 2021–2022. In this study, samples were collected from 9 different locations in Dhaka metropolitan city (Figure 1).

### 2.2 Samples selection and collection

Three different leafy greens viz., Coriander, (*Coriandrum sativum* L.) Mint (*Mentha* sp.), and Lettuce (*Lactuca sativa* L.) were selected which are frequently eaten raw (without cooking) that are considered ready‐to‐eat (RTE) products. A total of 27 working samples (9 for each leafy green) were collected in fresh and disease‐free conditions from 9 different supermarkets, local markets, and street markets (Table 1). Each sample was collected 3 times during each visit from the selected marketplaces and as a result, the data were obtained from a total of 81 samples of 27 working samples. During each visit, 100 grams of each sample were taken at random from each market. An insulated box with ice and a sterile zipper bag was used to collect all the samples, which were then thoroughly examined and analyzed within an hour of being collected. For further research, the collected samples were placed in sterile zipper bags and kept in the normal chamber of the refrigerator at 4–6°C.

**TABLE 1:** Detailed information on the collected leafy greens in this study.

Types of marketplaces	Location no.	Name of the marketplaces	Moisture condition of samples
Supermarket	1	Unimart	Dry
	2	Swapno	Dry
	3	Meena Bazaar	Dry
Local market	4	Rayer Bazaar	Moist
	5	Karwan Bazaar	Moist
	6	Mohammadpur Krishi Market	Moist
Street market	7	Dhanmondi street	Moist
	8	Zigatola street	Moist
	9	Mohammadpur street	Moist

### 2.3 Preparation of stock solutions

Within an hour of collection, the samples were taken into the laboratory, and prepared stock solutions were by submerging each 100 g sample with 1000 mL of distilled water, then diluted 5 folds in serial dilution by using a vortex mixture.

### 2.4 Isolation and identification of bacteria

A 0.05 mL of 1:10^−5^ diluted solutions were spread over the Nutrient Agar (NA) petri plates where each step was completed in triplicate and incubated for 24 h at 30°C. After 24 h, these overnight cultures in NA plates were observed where well‐distinct colonies with distinctive morphology were preferably selected and isolated on NA slants. Using streak plates, the chosen isolates were purified.

### 2.5 Biochemical tests

As per standard protocol of the Society of American Bacteriologists (1957), morphological and biochemical tests were done that were Gram‐staining, KOH solubility, Oxidase, Catalase, Motility, Simmon's citrate utilization, Casein hydrolysis, Starch hydrolysis, Gelatin liquefaction, and Levan production tests.

### 2.6 Culture on selective media

Four selective and differentiated media were used to identify bacterial species that were Eosin Methylene Blue (EMB) Agar, Salmonella Shigella (SS) Agar, Cetrimide Agar, and Bacillus cereus Agar. After culturing the isolates of the bacteria on these media, the growth and color of colonies were observed to identify bacteria.

### 2.7 Molecular analysis

The molecular detection was done at the Plant Pathology Laboratory using a combination of techniques including DNA extraction, DNA quantification, PCR amplification, gel electrophoresis and documentation, DNA purification, DNA sequencing, and bioinformatics analysis.

#### 2.7.1 Extraction of genomic DNA from pure cultures

Initially, 1 mL of overnight cultures were centrifuged (Model: Z‐216 M, HERMLE, Germany) at 15,000 × g for 2 min, and the supernatant was discarded, leaving behind the cell pellet. Next, the cells were resuspended in 480 μL of 50 mM EDTA, and 120 μL of lytic enzymes (lysozyme) was added, followed by incubation at 37°C for 60 min. The lysed cells were then centrifuged at 15,000 × g for 2 min, and the supernatant was discarded. For cell lysis, 600 μL of nuclei lysis solution was added, and the mixture was incubated at 80°C for 5 min, cooled to room temperature, and treated with 3 μL RNase solution at 37°C for 60 min. Protein precipitation was achieved by adding 200 μL of protein precipitation solution, vortexing vigorously at high speed for 30 s to mix the protein precipitation solution with the cell lysate. The vortex mixture was incubated on ice for 5 min and centrifuged at 15,000 × g* for 3 min. The resulting supernatants were transferred to another clean tube containing 600 μL of isopropanol, and DNA precipitation was facilitated by inverting the tube until visible DNA strands formed. After centrifugation at 15,000 × g* for 2 min the pellet cells were removed. Then, 600 μL of room temperature 70% ethanol was added and gently inverted the tubes several times to wash the DNA pellet and centrifuged at 15,000 × g* for 2 min. The DNA pellet was air‐dried for 15 min. Lastly, the DNA pellet was rehydrated in 100 μL of rehydration solution and incubated overnight at 4°C. Finally, the genomic DNA was stored at 20°C until use.

#### 2.7.2 DNA quantification

Using the NanoDrop Spectrophotometer (Model: ND2000, Thermo Scientific, USA), the purity and concentration of the extracted DNA were determined through 260/280 nm absorbance measures (Desjardins & Conklin, 2010).

#### 2.7.3 PCR amplification

The 16S ribosomal RNA (rRNA) gene was amplified using the bacteria‐specific primer 27F (5′‐AGAGTTGATCCTGGCTCAG‐3′) and universal primer 1492R (5′‐GGTTACCTTGTTACGACTT‐3′) which produced 1465‐bp amplicons (Wang et al., 2020). A 25 μL PCR mixture contained 12.5 μL of GoTaq™ G2 hot start master mix, 1 μL T DNA (25–65 ng/μL), 1 μL Primer 27F (10–20 pMol), 1 μL Primer 1492R (10–20 pMol), and 9.5 μL nuclease‐free water (Galkiewicz & Kellogg, 2008). The PCR assay was performed at a volume of 25 μL in an automated thermal cycler (Gene Atlas, Model: G2). The PCR thermal protocol consisted of initial denaturation at 95°C for 5 min, followed by 30 cycles at 95°C for 30 s, 52°C for 45 s, and 72°C for 90 s, and a final extension step at 72°C for 10 min and finally hold at 4°C for overnight (Raji et al., 2008). 5 μL aliquot of each amplified PCR product was subjected to electrophoresis on 1% agarose gel. The PCR product was stored in a refrigerator at −20°C.

#### 2.7.4 Electrophoresis and gel documentation

In the gel electrophoresis, Agarose powder (Cat: V3125) was mixed with Tris Borate EDTA (TBE) buffer (Cat: V4251) and heated to 80°C for 5 min to ensure complete dissolution, and then, ethidium bromide (Cat: H5041) was added for binding of the molecule to the DNA (Raji et al., 2008). The gel was poured onto a Horizontal Gel Electrophoresis (Model: Mini, CBS Scientific, USA) gel tray. The DNA samples, along with a 1 kb ladder (Cat: G754B) as a size marker, were loaded into the wells. Electrophoresis was run at 90 volts for 30 min to separate the DNA fragments based on their sizes. After the electrophoresis, the gel was transferred to an Alpha Imager Gel Documentation System (Model: Mini, Protein Simple, USA) for visualization and imaging of the DNA bands.

#### 2.7.5 DNA purification

DNA bands excised from the gel were dissolved using Membrane Binding Solution. Similarly, PCR amplification products were mixed with this solution. The DNA solutions were then transferred to SV Minicolumns for binding and subsequently washed using Membrane Wash Solution with ethanol. After ethanol evaporation, elution was performed by transferring Minicolumns to clean tubes and adding nuclease‐free water, and centrifuged. The purified DNA was obtained and ready for downstream applications.

#### 2.7.6 DNA sequencing

The purified DNA samples were sent to Bioneer (Seoul, Korea) for partial sequencing. The DNA sample was subjected to Sanger sequencing analysis. After getting the Sanger sequences, Chromas 2.6 software was used to generate the FASTA file containing the partial sequence of the 16S rRNA gene.

#### 2.7.7 DNA analysis

The sequences were analyzed by using the BLAST (Basic Local Alignment Search Tool) on the NCBI website (https://www.ncbi.nlm.nih.gov) for matching with existing nucleotide sequences in the NCBI GenBank database and obtained accession numbers. Phylogenetic analysis was performed using MEGA 11 (Molecular Evolutionary Genetics Analysis) software, where the maximum likelihood algorithm was selected to construct the phylogenetic tree with 1000 bootstraps and nucleotide Tamura‐Nei substitution model.

### 2.8 Estimation of bacterial load

After 24 h of inoculation, colony‐forming units (CFU) were first counted and recorded on the same day. At first, CFU was estimated for each mL of stock solution after that this value was multiplied by 10 (100 g samples submerged in 1000 mL stock solutions) to determine the CFU per gram of leafy green samples by following the equation:

$$\mathrm{CFU}/\mathrm{mL}=\frac{\mathrm{No}\;\text{of colonies}\;\mathrm{per}\;\text{plate}\times \text{Total dilution factor}}{\text{Volume of culture}\;\mathrm{per}\;\text{plate in}\;\mathrm{mL}}$$

Here, Total dilution factor = 10^5^ (Five‐fold serial dilution was performed). Volume of culture per plate = 0.05 mL.

CFU/g=CFU/mL×10.

### 2.9 Estimation of bacterial incidence

Measurement of the incidence of different types of bacteria in leafy greens was determined by the following equation:

$$\text{Incidence of bacteria}\;\left(\%\right)=\frac{\mathrm{No}.\;\text{of samples contaminated}\;\mathrm{by}\;\text{bacteria}\;}{\text{Total}\;\mathrm{no}.\;\text{of samples}}\times 100$$

### 2.10 Statistical analysis

The laboratory study was performed following Complete Randomized Design (CRD). The data were analyzed using the computer‐based software Statistix‐10. Differences in the means were statistically analyzed using ANOVA at a confidence level of 95% (*p* ≤ .05).

## 3 RESULTS AND DISCUSSION

### 3.1 Bacterial colony‐forming units' count

**FIGURE 2:**
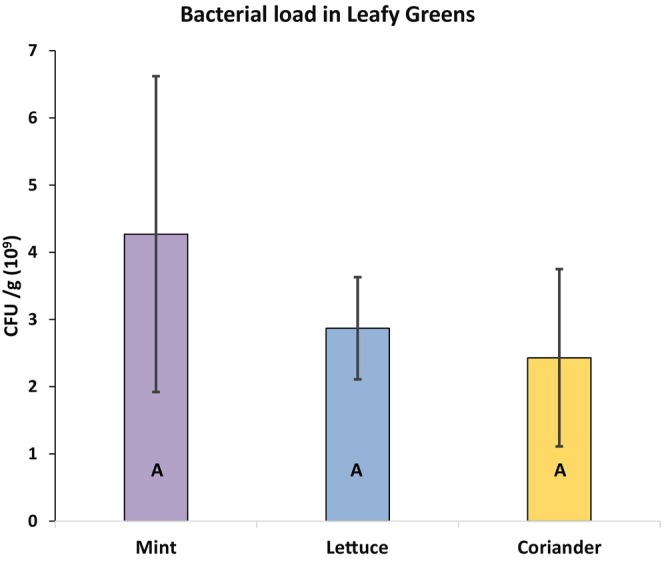
Mean colony‐forming units of bacteria isolated from per gram of mint, lettuce, and coriander leaves. Columns with a similar uppercase letter showed no significant differences (*F*‐test, *p* = .0603 > .05) among the mean bacterial load (Tukey test, *n* = 9).

The present study investigates the microbial contamination levels in three selected leafy greens viz. coriander, lettuce, and mint collected from 3 different types of marketplaces. Among the three leafy greens, the highest average (*n* = 9) bacterial load was observed in the mint leaves (4.27 ± 2.35 × 10^9^ CFU/g) similar to lettuce leaves (2.87 ± 0.76 × 10^9^ CFU/g), whereas the lowest was in the coriander leaves (2.43 ± 1.32 × 10^9^ CFU/g). Despite observable differences in mean counts, the results did not reach statistical significance (*p* = .0603) at a 95% confidence level, suggesting that the microbial populations in these leafy greens were not significantly different from each other (Figure 2).

**FIGURE 3:**
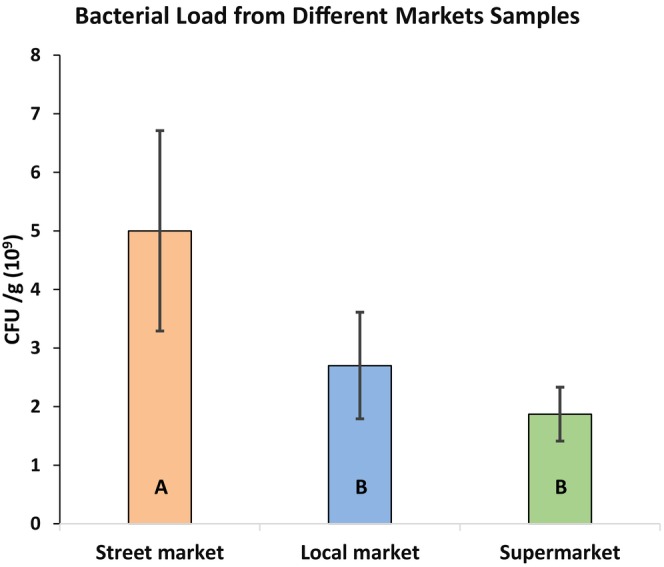
Mean CFU of bacteria isolated from per gram of different marketplace samples of leafy greens. Columns with different uppercase letters showed significant differences (*F*‐test, *p* = .0000 < .05) among the mean bacterial load (Tukey test, *n* = 9).

In terms of different marketplaces, the street markets samples showed the highest bacterial load (5.0 ± 1.72 × 10^9^ CFU/g) with statistically significant differences (*p* = .0000) compared to the supermarket samples (1.87 ± 0.46 × 10^9^ CFU/g) and the local market samples (2.7 ± 0.91 × 10^9^ CFU/g) where the supermarket and local market samples showed no statistically significant differences between them at a 95% confidence level (Figure 3). These findings suggest that market type significantly influences microbial populations in the samples, possibly due to varying handling practices or environmental conditions. Compared with other research findings, Yafetto et al. (2019) reported a bacterial load of lettuce leaves (1.23 × 10^8^ CFU/mL) in Cape Coast, Ghana. Houngla et al. (2019) studied selected leafy greens in Porto‐Novo, Republic of Benin, and found the highest total coliform counts in lettuces (3.21 × 10^3^ CFU/g). Nipa et al. (2011) observed viable bacterial colonies in coriander leaves ranging from 5.87 × 10^5^ to 1.8 × 10^6^ CFU/g and in peppermint leaves ranging from 2.2 × 10^5^ to 7.7 × 10^5^ CFU/g in Chittagong, Bangladesh.

The current results indicate a very high frequency of bacteria in all leafy greens samples, highlighting the unhygienic conditions in Dhaka city's markets where it crosses the acceptable level of bacterial load (>10^6^ CFU/g) for consumption of the leafy greens in raw condition according to the guidelines provided by Santos et al. (2005). These guidelines can serve as valuable references for ensuring food safety in the production and distribution of leafy green salads.

### 3.2 Isolation and identification of bacteria

In this study, different cultural, morphological, and biochemical tests were analyzed and identified bacterial genera following Bergey's Manual of Systematic Bacteriology Vol. I (Krieg & Holt, 1984) and Vol. II (Sneath et al., 1986). A total of 30 isolates of bacteria were obtained from which 9 genera of bacteria were initially detected including *Escherichia, Shigella, Klebsiella, Enterobacter, Bacillus, Pseudomonas, Staphylococcus, Vibrio*, and *Neisseria*.

*Staphylococcus aureus* was the only species identified through cultural, morphological, and biochemical tests. On the other hand, four selective and semi‐selective media were applied and identified *E. coli, Klebsiella pneumoniae, and Enterobacter aerogenes* in EMB Agar, *Pseudomonas aeruginosa* in Cetrimide agar, *Bacillus cereus* in Bacillus Cereus Agar and *Shigella* spp. in SS Agar. Besides, molecular analysis was done and identified *Bacillus subtilis, Bacillus velezensis*, and *Bacillus altitudinis*. From this study, a total of 12 bacteria species were identified from these 9 genera isolated from collected leafy green samples of different markets (Table 2).

**TABLE 2:** List of identified bacteria from collected leafy green samples.

Samples name	Types of marketplaces	Isolate no.	Identified bacteria
Coriander leaves	Supermarket	1	*Escherichia coli*
		2	*Klebsiella pneumoniae*
		3	*Bacillus cereus*
		4	*Pseudomonas aeruginosa*
		5	*Vibrio* sp.
	Local market	6	*Enterobacter aerogenes*
		7	*Bacillus. Subtilis*
		8	*Bacillus cereus*
	Street market	9	*Pseudomonas aeruginosa*
		10	*Bacillus cereus*
		11	*Escherichia coli*
		12	*Neisseria* sp.
		13	*Bacillus altitudinis*
Mint leaves	Supermarket	14	*Staphylococcus aureus*
		15	*Bacillus cereus*
	Local market	16	*Escherichia coli*
		17	*Bacillus velezensis*
		18	*Bacillus cereus*
	Street market	19	*Bacillus cereus*
		20	*Pseudomonas aeruginosa*
Lettuce leaves	Supermarket	21	*Bacillus cereus*
		22	*Neisseria* sp.
		23	*Klebsiella pneumoniae*
	Local market	24	*Enterobacter aerogenes*
		25	*Escherichia coli*
		26	*Neisseria* sp.
		27	*Bacillus cereus*
	Street market	28	*Escherichia coli*
		29	*Pseudomonas aeruginosa*
		30	*Shigella* sp.

The results of this study align with previous research conducted by other researchers in this field. The Centers for Disease Control and Prevention (2022) reported the presence of *E. coli* in leafy greens. Additionally, Andrea et al. (2022) identified *Vibrio cholerae* from salads vegetables in their investigation, while Sowmya et al. (2021) detected *Klebsiella pneumoniae, Enterobacter cloacae*, and *Pseudomonas aeruginosa* from different herbs. Moreover, Balali et al. (2020) and Turki et al. (2020) separately reported the occurrence of *E. coli, Bacillus* sp., *Pseudomonas* sp., *Shigella* sp., *Staphylococcus* sp., *Vibrio* sp., *Klebsiella* sp., *Neisseria* sp., *Enterobacter* sp., and other bacteria in fresh fruits and leafy greens.

In this study, the absence of certain bacteria like *Salmonella* spp., *Listeria monocytogenes*, and *Campylobacter jejuni* were identified by Balali et al. (2020) may be due to geographic variability, seasonal variations, limited sample sizes, and the focus of the studies on other bacterial species.

However, the present study focused on the identification of human pathogenic bacteria present in coriander, mint, and lettuce leaves. Among the 12 identified species of bacteria, this study confirmed the presence of 7 human pathogenic bacteria in these leafy green samples which were *B. cereus, E. coli, P. aeruginosa, K. pneumoniae, E. aerogenes*, *S. aureus*, and *Shigella* spp. The presence of these pathogenic bacteria raises significant concerns about the potential risks they pose to public health. These seven bacteria were reported as human pathogenic bacteria in several pieces of literature. For example, Yu et al. (2019) reported that *Bacillus cereus* is a foodborne opportunistic pathogen that can induce diarrheal and emetic symptoms. Additionally, *E. coli* and *Shigella* spp. have been responsible for numerous human deaths worldwide (Inderbinen & Stephan, 2016; Tomás‐Callejas et al., 2011). Mitov et al. (2010) highlighted that *Pseudomonas aeruginosa* is an opportunistic pathogen and one of the main bacteria causing nosocomial infections in hospitals, particularly affecting immunocompromised individuals. Furthermore, Zhang et al. (2018) indicated that *Klebsiella pneumoniae* is not only a major hospital‐acquired pathogen but also an important foodborne pathogen, capable of causing septicemia, liver abscesses, and diarrhea in humans. *Enterobacter aerogenes* is multi‐drug resistant to antibiotics and can be involved in urinary tract, gastrointestinal, and bloodstream infections, posing a potential risk of adult meningitis (Pradel & Pages, 2002). The study conducted by Kirk et al. (2014) sheds light on the significant impact of Enterotoxin‐producing *Staphylococcus aureus* as a causative agent of toxin‐mediated food poisoning. This identification adds to the growing body of knowledge concerning the microbial diversity associated with leafy greens and their potential implications for food safety and public health.

### 3.3 Study on the cultural characteristics

**FIGURE 4:**
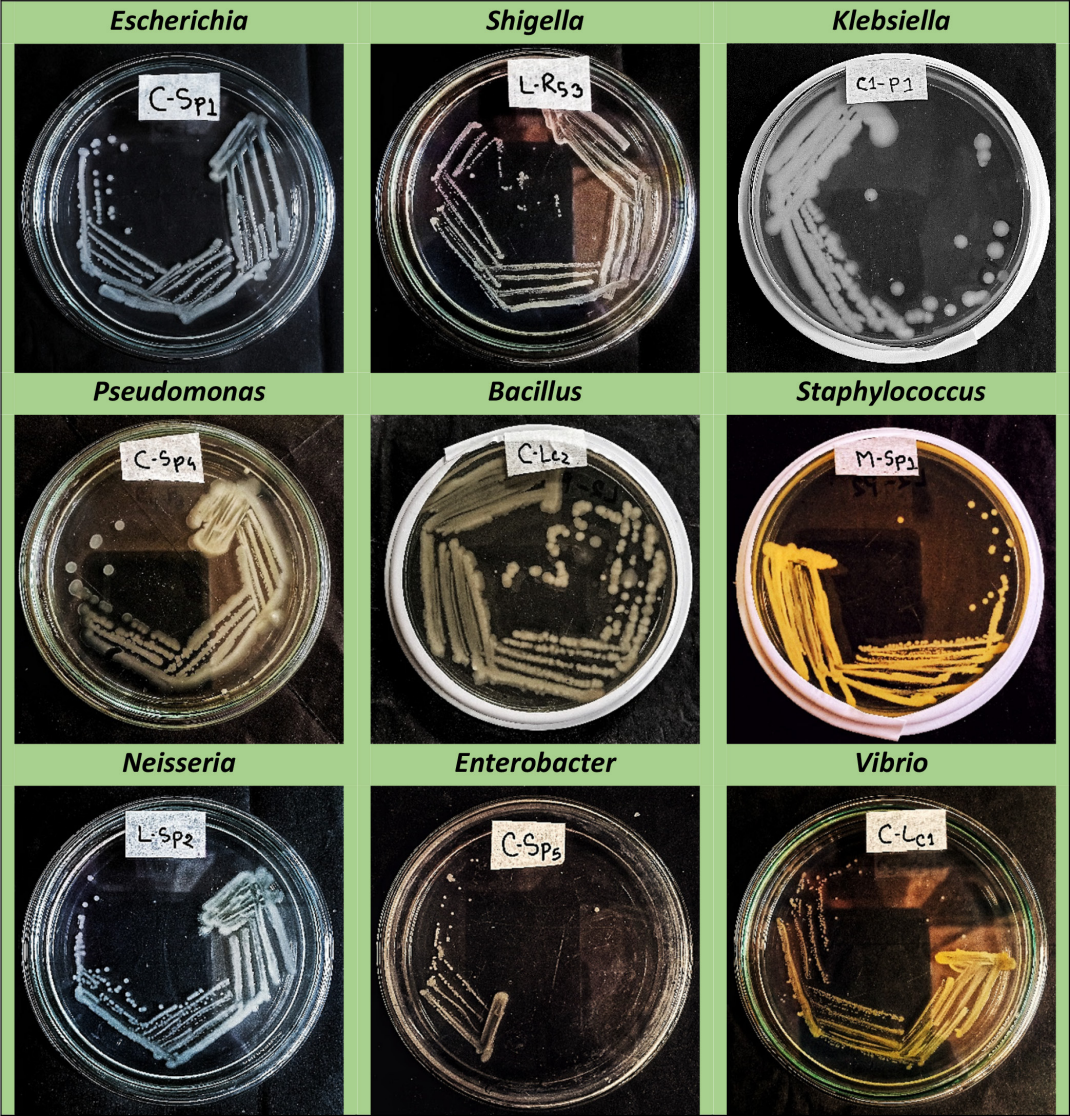
Growth of pure cultures on NA plates of the identified bacterial genera.

At first, the pure colony growths of different isolated bacteria were obtained by culturing them on the nutrient agar plate through streaking methods (Figure 4). The cultural characteristics like shape, size, margin, pigment, elevation, and texture of the bacterial pure cultures were examined to identify bacterial genera initially. The observed findings of the bacterial colony characters are presented in (Table 3).

**TABLE 3:** Cultural characteristics of identified bacteria on NA plates.

Bacteria	Size	Pigment	Shape	Margin	Elevation	Texture
*Escherichia*	Small‐ Medium	Grayish white	Round	Even	Convex	Smooth, Translucent, Mucoid
*Shigella*	Small	Grayish white	Round	Even	Convex	Smooth, Translucent, Mucoid
*Bacillus*	Medium	White	Round	Jagged	Convex or Flat depressed	Smooth or rough, Opaque, Dry
*Klebsiella*	Medium‐Large	White	Round	Even	Raised	Rough, Opaque, Mucoid
*Pseudomonas*	Small‐ Medium	Creamy	Round	Even	Flat	Rough. Opaque, Dry
*Staphylococcus*	Small	Golden‐yellow	Round	Even	Raised	Smooth, Opaque, Dry
*Enterobacter*	Small	Creamy	Irregular	Even	Raised	Smooth, Translucent, Mucoid
*Neisseria*	Small	Gray	Round	Even	Convex	Smooth, Opaque, Dry
*Vibrio*	Small	Gray	Round	Even	Raised	Smooth, Translucent, Mucoid

### 3.4 Study on the morphological characteristics

**FIGURE 5:**
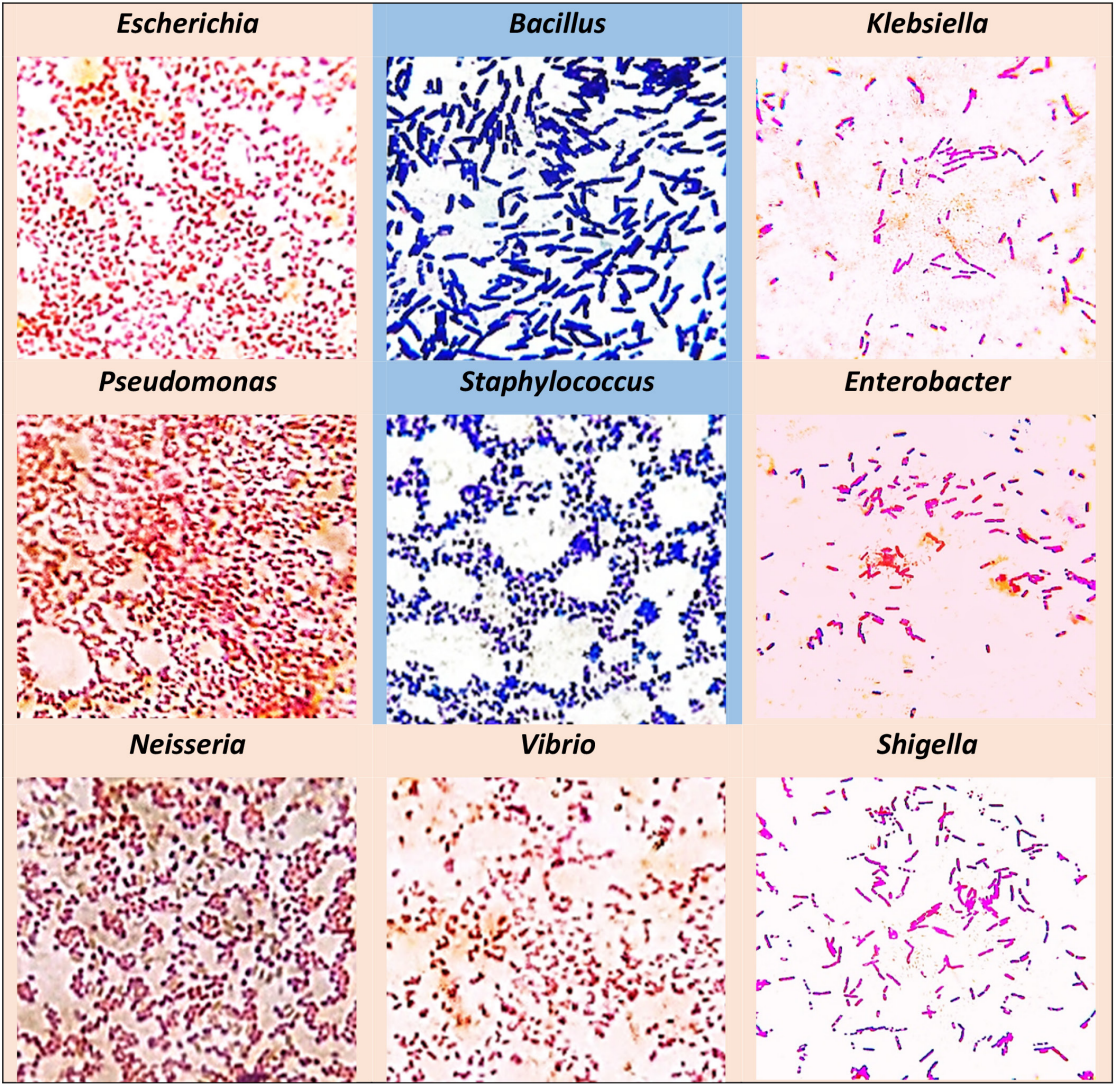
Cell morphology of the identified bacteria in gram staining test under a compound microscope (100×).

A comprehensive study of the morphological characteristics of the identified bacteria was performed after Gram staining and subsequent observation under a compound microscope. Among the identified bacteria, *Escherichia* and *Pseudomonas* showed small rod‐shaped; *Bacillus, Klebsiella, Shigella*, and *Enterobacter* rod‐shaped; *Vibrio* coma‐shaped; *Neisseria* bean/road‐shaped and *Staphylococcus* spherical‐shaped (Figure 5). These findings combined with results from cultural characterization, strengthen the elementary identification of the bacterial genera.

### 3.5 Study on the biochemical analysis

The biochemical tests conducted on the different isolates of bacteria revealed significant variations in the biochemical characteristics. Among the 30 isolates of bacteria, 60% were Gram‐negative bacteria which means they have a distinctive double membrane structure, and due to this structure, Gram‐negative bacteria are more resistant than Gram‐positive bacteria and cause significant morbidity and mortality worldwide (Breijyeh et al., 2020). The motility test displayed a positive result in 67% of the isolates, indicating the presence of motile bacteria. Moreover, the starch hydrolysis and casein hydrolysis tests exhibited positive outcomes in 58% and 67% of the isolates, respectively, indicating the ability of certain bacteria to break down these substrates. The gelatin liquefaction test showed positive results in 50% of the isolates, suggesting the presence of gelatinase‐producing bacteria. Interestingly, all isolates exhibited a positive reaction in the catalase test, indicating the presence of catalase‐producing bacteria in every case. On the other hand, only 50% of the isolates displayed a positive result in the oxidase test, signifying the presence of oxidase‐producing. The levan production test and citrate utilization test revealed positive results in 33% and 67% of the isolates, respectively, suggesting that certain bacteria could produce levan and utilize citrate as a carbon source. The findings of 10 biochemical tests are shown in (Table 4). These diverse results of biochemical analysis highlight the heterogeneity of the bacterial populations present in the leafy greens.

**TABLE 4:** Analysis of different biochemical test results of selected isolates for the identification of different bacteria.

Bacteria	Gram staining	KOH solubility	Motility	Starch hydrolysis	Casein hydrolysis	Gelatin liquefaction	Catalase	Oxidase	Levan production	Citrate utilization
*Bacillus cereus*	+	−	+	+	+	−	+	−	−	+
*Bacillus subtilis*	+	−	+	+	+	+	+	+	+	+
*Bacillus velezensis*	+	−	+	+	+	−	+	+	+	−
*Bacillus altitudinis*	+	−	+	+	−	+	+	+	+	−
*Escherichia coli*	−	+	+	−	−	−	+	−	−	−
*Pseudomonas aeruginosa*	−	+	+	−	+	+	+	+	+	+
*Neisseria* spp.	−	+	−	+	+	+	+	+	−	+
*Klebsiella pneumoniae*	−	+	−	−	+	−	+	−	−	+
*Enterobacter aerogenes*	−	+	+	+	+	−	+	−	−	+
*Shigella* spp.	−	+	−	−	−	−	+	−	−	−
*Staphylococcus aureus*	+	−	−	−	+	+	+	−	−	+
*Vibrio* spp.	−	+	+	+	−	+	+	+	−	+

### 3.6 Study of the bacterial cultures on selective media

**FIGURE 6:**
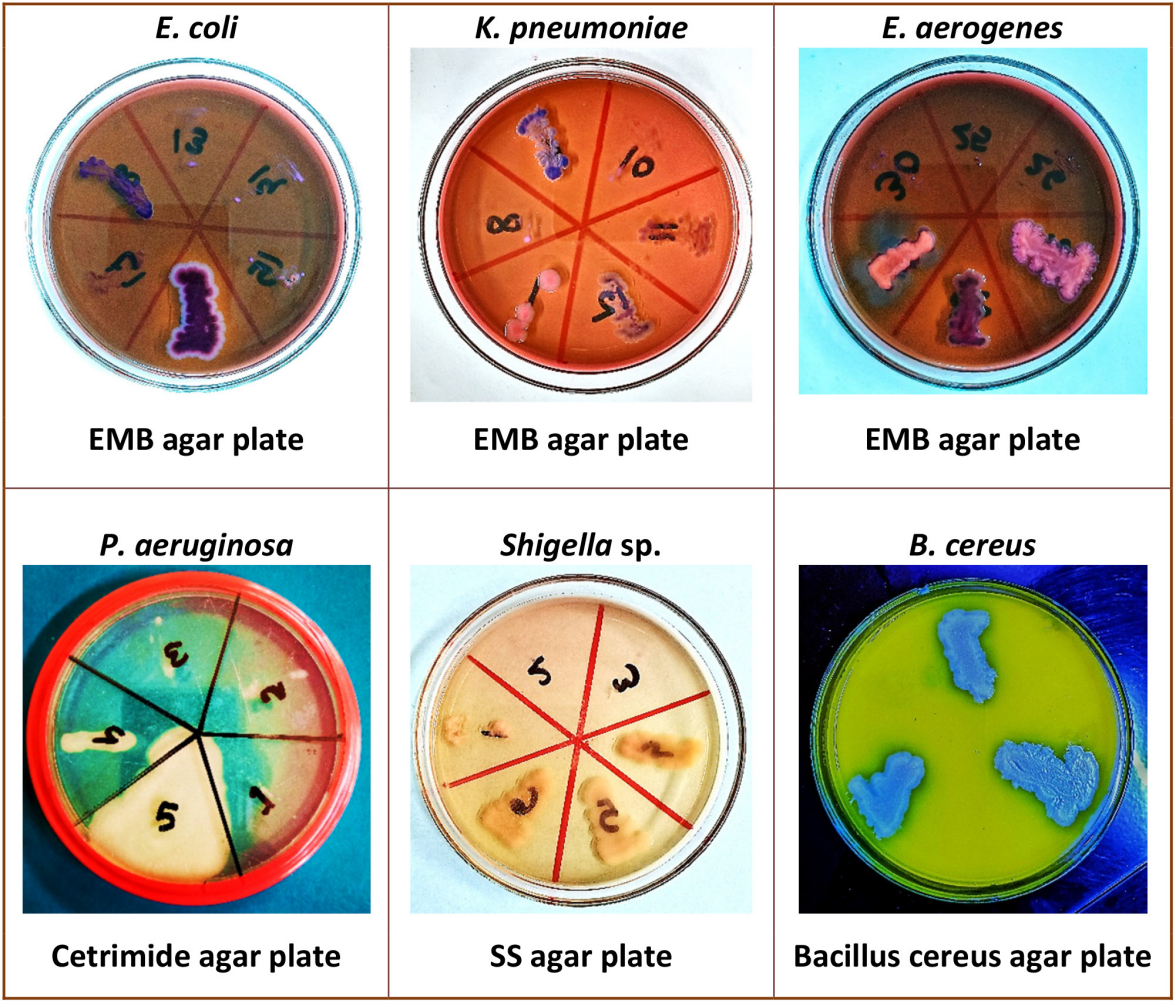
Growth of different bacteria on four selective and semi‐selective agar plates.

By observing the appearance of bacterial colonies on four different selective and semi‐selective (differentiated) media, 6 different bacteria were identified and confirmed (Figure 6). *E. coli* showed a blue colony without a metallic green sheen, *Klebsiella pneumoniae* showed a pink mucoid colony, and *Enterobacter aerogenes* showed a pink dull colony on EMB agar. These color variations are indicative of lactose fermentation, with each bacterium showing a unique color response due to the differential properties of the EMB agar. Aryal (2022a) corroborated these findings, explaining that EMB agar is a differential medium that can inhibit the growth of Gram‐positive bacteria while aiding in distinguishing lactose fermenters based on colony colors. The results from this study align with Aryal's description of the EMB agar's capabilities.

*Pseudomonas aeruginosa* showed a cream colony on Cetrimide Agar. This selective medium is known for its ability to isolate and identify *Pseudomonas* species, particularly *Pseudomonas aeruginosa*, due to its characteristic growth and pigment production. The observed cream colony appearance is consistent with previous research on the distinctive colonial appearance of *Pseudomonas aeruginosa* on Cetrimide Agar (Brown & Lowbury, 1965).

*Bacillus cereus* exhibited a blue colony on Bacillus cereus agar. Holbrook and Anderson (1980) supported these findings, reporting that Bacillus cereus agar is a selective medium specifically designed for the identification of *Bacillus cereus*. The peacock blue color produced by *Bacillus cereus* on this agar medium is considered a distinguishing feature of the species.

*Shigella* sp. produced a transparent colony on SS Agar. SS agar is a semi‐selective medium that inhibits the growth of most Gram‐positive bacteria while allowing the growth of Gram‐negative enteric pathogens like *Shigella* and *Salmonella*. The transparent colony appearance aligns with the reported colorless characteristics of *Shigella* spp. on SS agar as described by Aryal (2022b).

### 3.7 Study on molecular analysis

#### 3.7.1 Gel electrophoresis of PCR products

**FIGURE 7:**
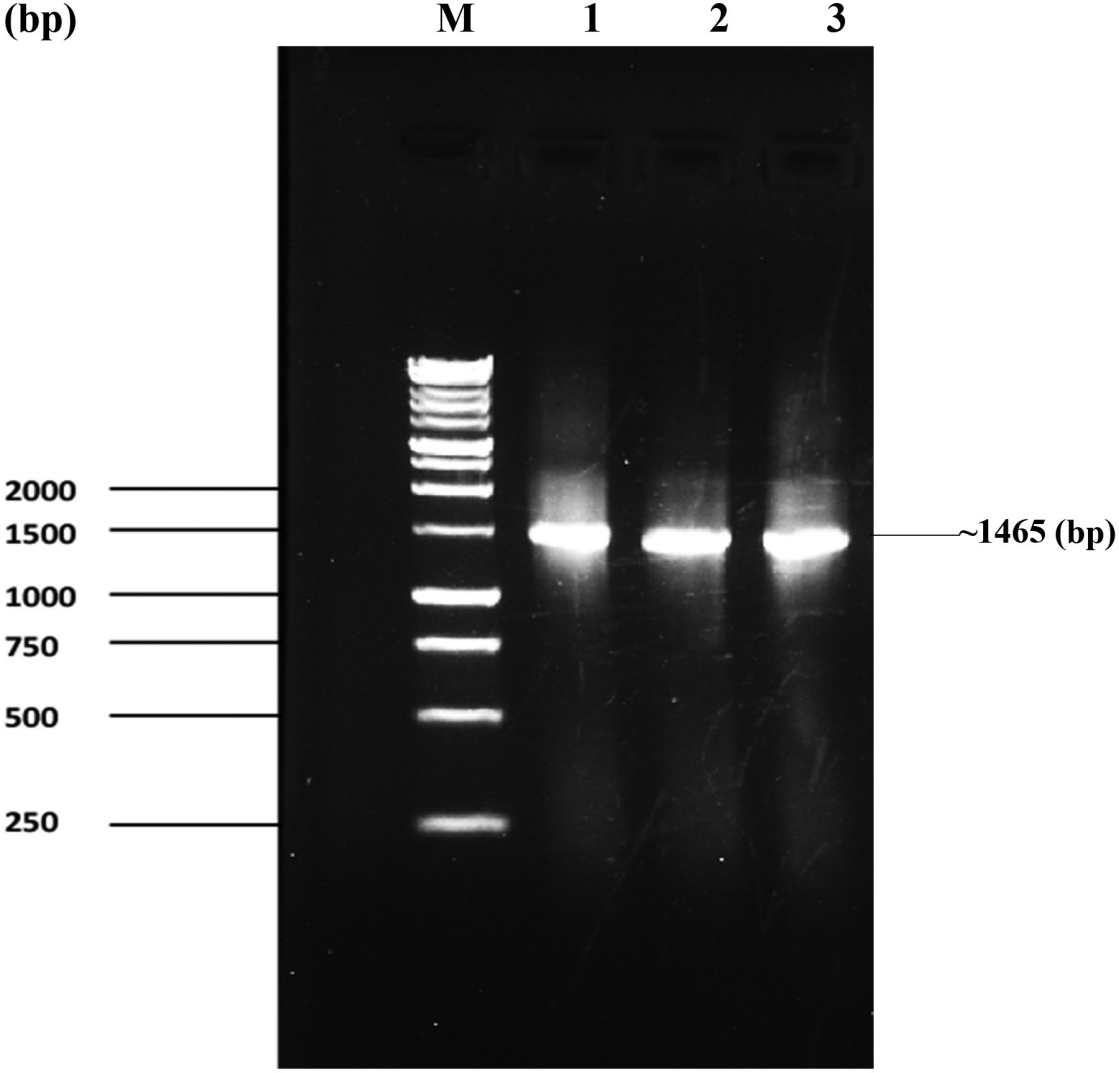
PCR amplified product from (1) *Bacillus subtilis*, (2) *Bacillus velezensis*, and (3) *Bacillus altitudinis* isolates of bacteria. M: denotes 1 kb DNA ladder (Marker).

The Gel‐Doc system of PCR products using primers 27F and 1492R resulted in the amplification of a DNA band with a size of approximately 1465 base pairs (bp). The obtained DNA band was very close in size to the target band, indicating successful PCR amplification. A Bench Top 1 kb DNA ladder was used as a size marker, confirming the size of the amplified DNA fragment. Lanes 1, 2, and 3, (Figure 7) represent the bacterial strains of *B. subtilis, B. velezensis*, and *B. altitudinis*, respectively, which exhibited the amplified DNA band. The results suggest that all 3 bacteria isolates contain the targeted DNA region, as indicated by the presence of the amplified band at the expected size. The Gel‐Doc system confirms the specificity and successful amplification of the desired DNA fragment in the bacterial samples.

The results demonstrate a consistent detection of an approximately 1465 ± bp DNA band in the gel doc system, which aligns with Nurfitri et al. (2019) findings. This similarity is observed when using 27F and 1492R primers along with the 1 kb DNA ladder as a marker. The presence of this band indicates that the used primers and PCR conditions were amplified well in accordance with the primary target of the 16S rRNA gene.

#### 3.7.2 Analysis of DNA sequences

The sequences were analyzed by using the BLAST on the NCBI website (https://www.ncbi.nlm.nih.gov) for matching with existing nucleotide sequences in the NCBI GenBank database. The partial sequences of 16S rRNA of isolated bacteria were submitted to NCBI for deposition in the GenBank and obtained accession number (Table 5).

**TABLE 5:** Results of bacterial 16S rRNA gene identification with BLAST Program.

LAB strain			BLAST alignment				
Sl. no.	Identified bacterial isolates	Obtained accession no.	Species	Query coverage	E value	Percent identity	Accession no.
1.	*Bacillus subtilis* strain SAUBD‐B1	OQ740285.1	*Bacillus subtilis* strain Cu31	99%	0.0	100.00%	KY085997.1
2.	*Bacillus velezensis* strain SAUBD‐B2	OQ740286.1	*Bacillus velezensis* strain ND06	100%	0.0	99.75%	ON386277.1
3.	*Bacillus altitudinis* strain SAUBD‐B3	OQ740287.1	*Bacillus altitudinis* strain LY43	99%	0.0	99.83%	MZ067888.1

It has been observed that isolated *Bacillus subtilis* strain SAUBD‐B1 (Accession no. OQ740285.1) showed 100% identity to the *Bacillus subtilis* strain Cu31 16 s ribosomal RNA gene with accession no. KY085997.1. *Bacillus velezensis* strain SAUBD‐B2 (Accession no. OQ740286.1) showed 99.75% identity to the *Bacillus velezensis* strain ND06 16 s ribosomal RNA gene with accession no. ON386277.1. Lastly, *Bacillus altitudinis* strain SAUBD‐B3 (Accession no. OQ740287.1) showed 99.83% identity to the *Bacillus altitudinis* strain LY43 16 s ribosomal RNA gene with accession no. MZ067888.1. According to Pangastuti (2006), the identity values exceeding 97% demonstrate that the species discovered in the samples is not a novel species and is more identical to those found in the GenBank.

#### 3.7.3 Analysis of phylogenetic tree

**FIGURE 8:**
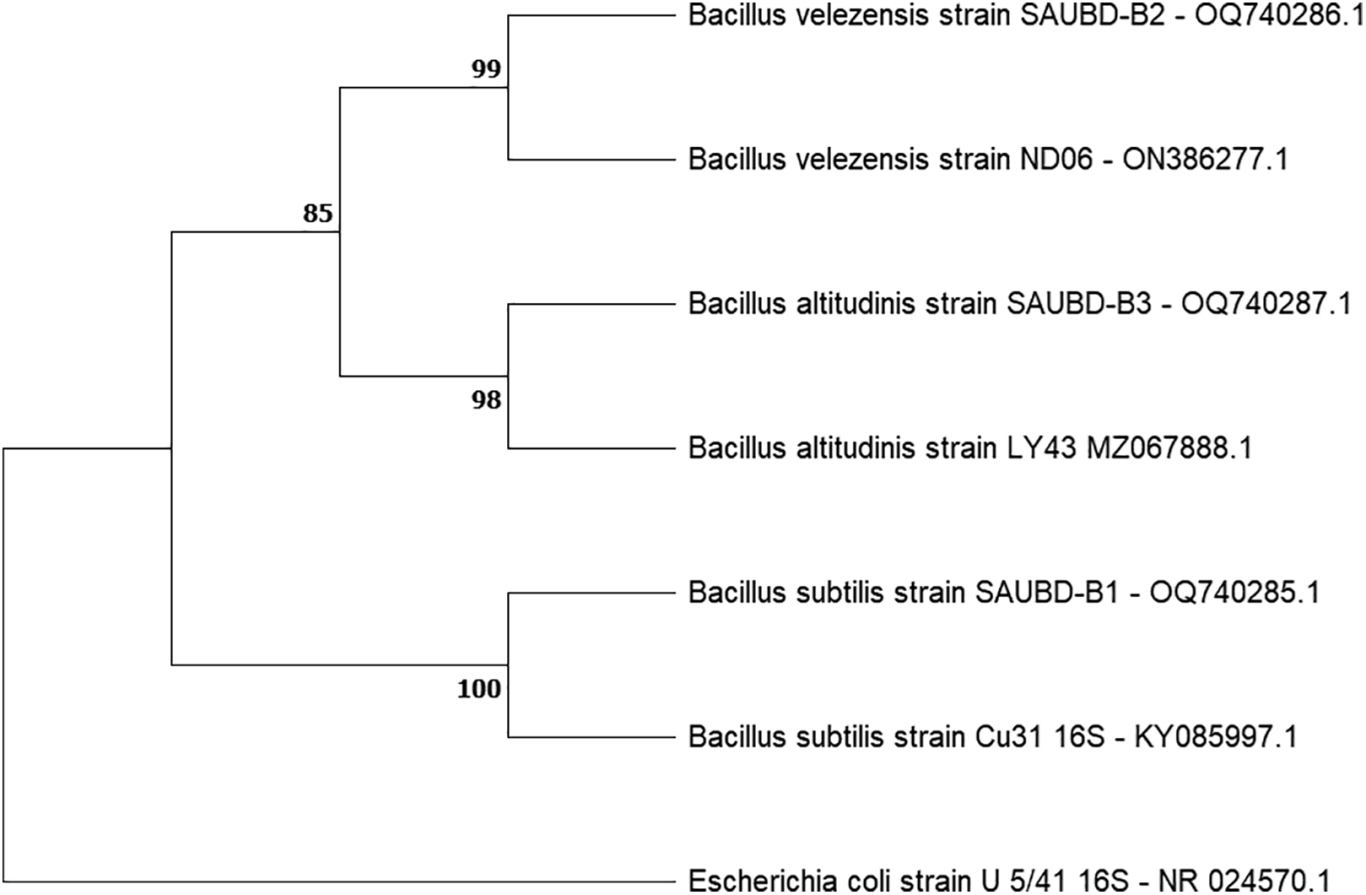
Analysis of phylogenetic tree of *Bacillus subtilis, Bacillus velezensis*, and *Bacillus altitudinis* strains based on 16S rRNA sequences. The numbers at the branches are bootstrap confidence percentages from 1000 bootstrap trees. The *Escherichia coli* strain U 5/41 NR_0245701 was used as the outgroup.

The phylogenetic analysis was performed using genetic sequences of *B. subtilis, B. velezensis*, and *B. altitudinis* strains. The sequences were aligned, and a maximum likelihood phylogenetic tree was constructed using MEGA 11 software. The tree was rooted using an outgroup sequence and bootstraps values were calculated to assess the statistical support for the branching patterns (Figure 8).

The resulting phylogenetic tree revealed the relationships among the studied strains. Notably, *B. subtilis* strain SAUBD‐B1 and *B. subtilis* strain Cu31 were found to form a single clade with strong bootstrap support. Similarly, *B. velezensis* strain SAUBD‐B2 and *B. velezensis* strain ND06; *B. altitudinis* strain SAUBD‐B3 and *B. altitudinis* strain LY43 were also grouped together in a well‐supported clade. The identification of shared clades indicates a close evolutionary relationship between certain strains, supporting the notion that they belong to the same species.

### 3.8 Assessment of bacterial incidence

**FIGURE 9:**
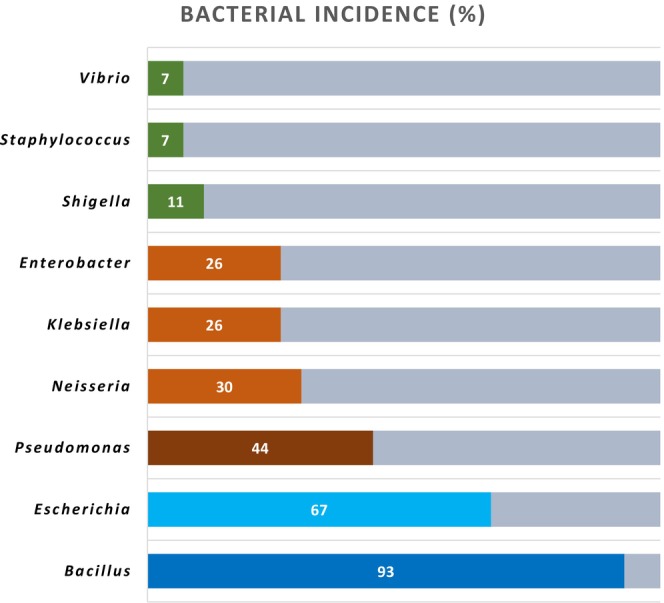
Bar chart showing the incidence of identified bacterial genera in leafy greens.

Among the 27 tested samples, 9 genera of bacteria were identified where *Bacillus, Escherichia, Pseudomonas*, *Neisseria*, *Klebsiella*, *Enterobacter*, *Shigella*, *Staphylococcus*, and *Vibrio* were present in 25, 18, 12, 8, 7, 7, 3, 2, and 2 no. of tested samples, respectively. The incidence of these genera of bacteria is shown in Figure 9.

Comparing these results with the findings of Srisamran et al. (2022), their study reported a higher prevalence of *E. coli* in coriander and lettuce reaching 100% in open‐market samples besides 83.3% and 91.75% in supermarket samples in coriander and lettuce, respectively. Additionally, *Salmonella* was present (12.5% in open market samples, absent in supermarket samples) in different market samples, and *Shigella* was absent in all samples. These variations in bacterial incidence highlight the importance of considering the source and handling practices of leafy greens in different marketplaces.

Turki et al. (2020) also investigated the prevalence of pathogenic bacteria in leafy greens. Their study reported a 100% incidence of *Staphylococcus, Bacillus, Enterobacter, Shigella*, and *Escherichia coli*. *Klebsiella* and *Salmonella* also showed a high incidence at 92%, and *Pseudomonas* were found in 80% and 72% of the samples, respectively. *Neisseria* had a lower incidence of 12%.

These collective findings emphasize the variability in bacterial incidences in leafy greens across different studies and regions. Further research is essential to investigate the contributing factors behind these variations and to identify effective strategies to mitigate bacterial contamination in leafy greens. Implementing strict food safety measures and raising awareness among producers, vendors, and consumers about proper hygiene practices can play a crucial role in reducing the risk of bacterial contamination in leafy greens and ensuring food safety.

## 4 CONCLUSION

The study aimed to detect potential human pathogenic bacteria in ready‐to‐eat leafy greens sourced from diverse marketplaces in Dhaka city. Across coriander, lettuce, and mint leaves, a diverse array of bacterial genera was identified. Notably, twelve distinct bacterial species were isolated, among them recognized human pathogenic bacteria were *B. cereus, E. coli, P. aeruginosa, K. pneumoniae, E. aerogenes, S. aureus*, and *Shigella* spp. All leafy greens collected from different marketplaces exhibited bacterial loads surpassing acceptable thresholds, signaling unsanitary conditions in Dhaka city's markets. The high prevalence of *Bacillus* and *Escherichia*, including pathogenic species like *B. cereus* and *E. coli* poses concerning implications for food safety. This underscores the urgent need for enhanced food safety measures throughout the production and distribution of ready‐to‐eat leafy greens within Dhaka city.

## AUTHOR CONTRIBUTIONS

**Raihan Ferdous:** Conceptualization (equal); data curation (equal); formal analysis (lead); investigation (lead); methodology (lead); project administration (equal); resources (equal); software (lead); supervision (equal); validation (equal); visualization (equal); writing – original draft (lead); writing – review and editing (equal). **Nazneen Sultana:** Funding acquisition (lead); methodology (supporting); supervision (equal); validation (equal); writing – review and editing (equal). **Md. Belal Hossain:** Conceptualization (equal); funding acquisition (supporting); project administration (equal); supervision (equal); validation (equal); writing – review and editing (equal). **Rifat Ara Sultana:** Formal analysis (supporting); methodology (supporting); validation (equal); visualization (equal); writing – review and editing (equal). **Sanzida Hoque:** Data curation (equal); validation (equal); visualization (equal); writing – review and editing (equal).

## FUNDING INFORMATION

No external funding was received to support this work.

## CONFLICT OF INTEREST STATEMENT

The authors have no conflict of interest to declare that are relevant to the content of this article.

## Data Availability

The data that support the findings of this study are available on request from the corresponding author. The data are not publicly available due to privacy.
